# Continuous Renal Replacement Therapy in Pediatric Patients With Acute Kidney Injury After Liver Transplantation

**DOI:** 10.3389/fped.2022.878460

**Published:** 2022-06-22

**Authors:** Yan Sun, Sinan Gao, Xingqiang Wang, Lixin Yu, Min Xu, Wei Gao, Chao Sun, Bing Wang

**Affiliations:** ^1^Organ Transplantation Center, Tianjin First Central Hospital, Tianjin, China; ^2^Tianjin Key Laboratory for Organ Transplantation, Tianjin, China

**Keywords:** liver transplantation, infants and young children, acute kidney injury, continuous renal replacement therapy, postoperative mortality

## Abstract

**Objective:**

This study aimed to explore the clinical application of continuous renal replacement therapy (CRRT) in pediatric patients with acute kidney injury (AKI) after liver transplantation.

**Methods:**

Pediatric patients who underwent liver transplantation were retrospectively investigated. Those who developed AKI within 1 year after the surgery were included and divided into a CRRT group and a non-CRRT group. The perioperative conditions and postoperative complications of the two groups were compared along with the prognoses of the groups to analyze the high-risk factors of the postoperative CRRT.

**Results:**

189 (36.91%) patients developed AKI within 1 year after the liver transplantation surgery. There were 18 patients in the CRRT group and 171 in the non-CRRT group. The differences in the preoperative conditions were not statistically significant between the two groups. Compared with the non-CRRT group, patients in the CRRT group had significantly longer transplantation times, higher volumes of intraoperative hemorrhage, and increased incidence of postoperative unscheduled surgery, postoperative primary nonfunction of the transplanted liver, secondary liver transplantation, hepatic artery occlusion, and intestinal fistula (*P* < 0.05). Moreover, the proportion of patients in AKI stage 3 is higher in the CRRT group (83.33%) than that in the non-CRRT group (11.11%), *P* < 0.001. The median time to initiate CRRT was 10 days postoperatively, the median number of CRRT treatments per patient was 2 times, the average duration of each CRRT treatment was 10.1 h, and the average rate of the decrease in blood creatinine per treatment was 25.6%. Results of multivariate logistic regression analysis showed that AKI stage 3 [OR=40.000, 95%CI (10.598, 150.969), *P* = 0.016], postoperative unscheduled surgery [OR=6.269, 95%CI (3.051, 26.379), *P* = 0.007], and hepatic artery occlusion [OR = 17.682, 95%CI (1.707, 40.843), *P* = 0.001] were recognized as risk factors for postoperative AKI with CRRT therapy. The one- and two-year survival rates were 72.22% and 72.22% in the CRRT group, respectively; and 97.08% and 96.49% in the non-CRRT group, accordingly. There were statistically significant differences in the one- and two-year survival rates between the two groups (*P* < 0.001).

**Conclusion:**

The incidence of AKI after liver transplantation in pediatric patients was high. Patients with AKI stage 3, hepatic artery occlusion, and underwent unscheduled surgery postoperatively were with a high likelihood of receiving CRRT, which was related to a lower one- and two-year survival rates. CRRT effectively improved the one- and two-year survival rates.

## Introduction

Liver transplantation is the largest abdominal surgery. The vast intraoperative hemodynamic fluctuations and sizeable intraoperative blood loss/transfusions involved have a significant impact on systemic circulation and renal perfusion, resulting in a high incidence of acute kidney injury (AKI) after liver transplantation. Most pediatric liver transplant recipients are infants and young children under the age of 3 years; their organ functions are not well developed, and they have poor compensatory capacity. Therefore, the incidence of AKI after liver transplantation in children is high, with a reported incidence of 40% to 70% (1). According to a statistical analysis by Wu Man et al. (2) of 112 children aged 5–24 months who underwent parental liver transplantation at the Organ Transplantation Center of the Tianjin First Central Hospital from January to December 2019, the incidence of postoperative AKI was 40.18%. Previous studies have shown that low preoperative serum creatinine level, high preoperative pediatric end-stage liver disease (PELD) score, severe myocardial depression in the early stage of reperfusion, and markedly decreased cardiac output and blood pressure postoperatively are independent risk factors for postoperative AKI (2).

Continuous renal replacement therapy (CRRT) is one of the most commonly used forms of blood purification, which can remove toxins and inflammatory medium and small molecule mediators, making it useful for treating renal failure, heart failure, volume overload, sepsis, multiple organ dysfunction syndromes (MODS), adult respiratory distress syndrome (ARDS), severe pancreatitis, and some immune diseases. However, there are few studies on the risk factors and prognosis of CRRT in pediatric patients with postoperative AKI after the liver transplantation.

This study retrospectively investigated the pediatric patients with postoperative AKI and CRRT after liver transplantation, analyzed their risk factors and the characteristics of the CRRT, and discussed the prognosis of the involved pediatric patients.

## Subjects and Methods

### Subjects

A retrospective analysis was conducted on pediatric patients who underwent liver transplantation at Tianjin First Central Hospital from January 1, 2019, to June 1, 2021, and developed AKI within 1 year after the surgery. According to the criteria for AKI established by the organization Kidney Disease: Improving Global Outcomes, those with an absolute increase in blood creatinine (Cr) of ≥26.5 mmol/L within 48 h, or an increase of ≥50% from the baseline, or a urine volume of <0.5 ml/(kg/h) (≥6 h, <12 h), were considered to be AKI stage 1. Those with an elevation in blood Cr of ≥2-fold from the baseline or a urine volume of <0.5 ml/(kg/h) (≥12 h) were considered to be AKI stage 2. Those with a blood Cr level of ≥353.6 mmol/L, or ≥ an elevation of 3-fold from the baseline, or a urine volume of <0.3 ml/(kg/h) (≥24 h) were considered to be AKI stage 3, in which case, renal replacement therapy could be initiated.

The exclusion criteria were pediatric patients who had preoperative renal insufficiency or had undergone renal replacement therapy preoperatively.

The present study conformed to the standards of the Ethics Committee of Tianjin First Central Hospital (approval number 2019N097KY). The guardians of the pediatric patients were informed, and written consents were obtained.

### Methods

Pediatric patients with postoperative AKI after the liver transplantation were divided into CRRT and non-CRRT groups according to whether the CRRT was conducted or not. Before the initiation of the CRRT treatment, no pediatric patients had undergone the liver bridging therapy such as molecular absorbent recirculating system (MARS), single-pass albumin dialysis (SPAD), and plasma separation.

The details of the CRRT are as follows:

#### The Initiation of the CRRT Therapy

Regardless of whether the patient was diagnosed with AKI stage 3 or not, The CRRT therapy was initiated when patients were with persistent deterioration of renal function after the liver transplantation, with the failure of active treatment of the potential causes, such as reversing systemic hypovolemia and renal hypoperfusion and replacing the use of nephrotoxic drugs, and with symptoms such as anuria, severe hyperkalemia, severe metabolic acidosis, acute pulmonary edema, lethargy, and disturbance of consciousness occurred for a consecutive 48-h.

#### Indwelling of a Double-Lumen Catheter for Blood Purification

The indwelling of double-lumen catheters was performed under ultrasound guidance. Low-dose sedatives and analgesics were administered before the catheter indwelling procedure to reduce agitation, pain, and stress during puncture. The left internal jugular vein was selected for the indwelling double-lumen catheter in most of the pediatric patients. A Campbell 6F or 8F double-lumen catheter was selected for puncture according to the height and weight of the pediatric patient.

#### Selection of Blood Purification Methods and Parameters

The mode of CRRT was decided based on the hepatic and renal function as well as the inflammatory response of the pediatric patients. If a patient was predominantly in renal failure, hemodialysis was the preferred method of dialysis to remove toxins with a small molecular weight. If the patient had hepatic dysfunction, a significant systemic inflammatory response, and was less hemodynamically stable, hemofiltration was considered to remove toxins and inflammatory mediators with small or medium molecular weights. Hemodiafiltration was an option if the pediatric patient had renal failure combined with sepsis and severe inflammatory response. A model IQ-21 (Asahi Kasei, Japan) dialysis machine, a CHDF-21P blood pipeline (Asahi Kasei, Japan), and an AEF-03 (Asahi Kasei, Japan) hemofilter were used. During treatment, the blood flow rate was 3–5 ml/kg^−1^/min^−1^, the total flow rate of the dialysis solution or replacement solution was 50–80 ml/kg^−1^/h^−1^, and the treatment time was 6–24 h.

#### Choice of Anticoagulation Treatments

If the child had no bleeding tendency with well-recovered coagulation function and the platelet count was >10^*^10^∧^9/L, 3–5 mg/h of heparin sodium was administered for anticoagulation. For those with no bleeding tendency, good coagulation recovery, and a platelet count of <10^*^10^∧^9/L, 1–2 mg/h of argatroban was administered for anticoagulation. If the patient had a bleeding tendency, a sodium citrate anticoagulant was administered in a local manner at a flow rate of 1.2–1.5 times the blood flow rate/h. During treatment, the international normalized ratio, kaolin partial thromboplastin time, activated clotting time, and calcium concentration in the arterial and post-filtered blood were monitored depending on the anticoagulant used.

#### Circulation Maintenance During Treatment

If the child weighed <10 kg or had hypotension or insufficient effective circulating blood volume, 10 g of albumin was administered to prefill the pipeline. The blood was drained slowly at a low rate, especially during the first 30 min of treatment, and the changes in vital signs were closely monitored. For those with underlying heart disease or heart failure symptoms, slow, little, or even no blood return was required at the end of treatment to prevent the induction of heart failure.

#### Observational Indexes of the Effect of Continuous Renal Replacement Therapy and Criteria for Discontinuation of Treatment

The patients' blood urea nitrogen, Cr, cystatin, B-type natriuretic peptide precursor, and blood gas analysis were monitored before and after each treatment. In cases of improved renal function, gradually increased urine volume, reduced inflammatory reaction, no obvious acidosis or hyperkalemia with stable circulation, and no obvious fluid overload performance together with gradually improved functions of the heart, liver, and other organs, the duration of each blood purification treatment was reduced and the interval of blood purification extended until the treatment was terminated.

#### The Timing of Temporary and Permanent Discontinuation of CRRT Therapy

There was no consensus or other common indications for determining when the CRRT should be discontinued temporarily or permanently, so the duration of each CRRT treatment and the total number of CRRT treatments were determined by the responsible pediatrician according to the clinical situation of the patient. The duration of each CRRT treatment plan was arranged for 6 to 12 h, and the final duration of each treatment was determined according to the tolerance of the child, the maintenance of the dialyzer, and the achievement of the primary treatment goals (reducing water load, alleviating acidosis, or treating hyperkalemia).

### Statistical Methods

The study used SPSS 19.0 software for data processing. For the measurement data, a normal distribution test was performed first. The normally distributed measurement data were expressed as mean ± standard deviation ($\bar{x}$ ± *s*), while those that failed to satisfy the normal distribution were expressed as Median (the minimal value ~ maximal value). The countable data were expressed as percentages (%). For comparison between groups, a *t*-test or rank-sum test for independent samples was used for the measurement data, and Fisher's exact probability test or Fisher's exact probability method was used for the categorical variables. The significant levels in all the statistical tests were set as *P* < 0.05. CRRT-related variables with statically significant differences were included in a multivariate logistic regression analysis to identify the risk factors by enter method, the results were expressed as [Odds Ratio (OR), 95% Confidence Interval (95%CI), *P* value], and variables with *P* < 0.05 were set as a statistically significant difference.

Postoperative mortality was set as the final event, the one- and two-year survival rates were calculated and compared, and the Kaplan–Meier estimator curve was plotted with the area under the curve (AUC) was calculated.

## Results

### The Occurrence of Acute Kidney Injury in Children After Liver Transplantation

Of the 512 pediatric cases with liver transplantation conducted at Tianjin First Central Hospital from January 1, 2019, to June 1, 2021, 189 cases (36.91%) developed AKI within 1 year, mostly within 2 weeks postoperatively (183/189, 96.83%), and gradually returned to preoperative levels within 1 month postoperatively (181/189, 95.77%). These pediatric patients with postoperative AKI were divided into a CRRT and a non-CRRT group according to whether they were treated with CRRT or not. The general preoperative and intraoperative conditions between the two groups of pediatric patients are shown in Table 1. There were no statistically significant differences in the preoperative characteristics between the two groups. Compared with the non-CRRT group, the CRRT group had longer operation time of the liver transplantation (7.5 ± 1.3 h vs. 8.8 ± 1.5 h, *P* < 0.001), higher volumes of intraoperative hemorrhage [310 (200~400) ml vs. 370 (220~800) ml, *P* = 0.045], and a higher proportion of patients in AKI stage 3 (11.11% vs. 83.33%, *P* < 0.001).

**Table 1:** Comparison of perioperative baseline characteristics between the CRRT group and non-CRRT group of pediatric patients with acute kidney injury.

**Items**	**The CRRT group (*N* = 18)**	**The non-CRRT group (*N* = 171)**	***P*** **value**
The average age (Month)	9.89 (4~35)	8.22 (5~36)	0.318
Gender (Male/female)	7/11	76/95	0.460
Body weight (kg)	12.8 ± 7.3	10.7 ± 5.5	0.250
The primary disease (BA) [n (%)]	15 (83.33)	139 (81.29)	0.832
PELD score	17 (10~33)	12 (8~35)	0.274
Preoperative congenital heart disease [n (%)]	2 (11.11)	21 (12.28)	0.885
The preoperative conduction of the Kasai biliary atresia surgery [n (%)]	11 (61.11)	103 (60.23)	0.942
Preoperative antibiotic exposure [n (%)]	13 (72.22)	135 (78.95)	0.510
The preoperative indwelling of central venous catheter [n (%)]	4 (22.22)	32 (18.71)	0.718
Preoperative plasma replacement therapy [n (%)]	4 (22.22)	27 (15.79)	0.483
The duration time of liver transplantation surgery (hours)	8.8 ± 1.5	7.5 ± 1.3	<0.001^*^
The average intra-operative hemorrhage (ml) [median (quartile)]	367.2 ± 96.1	274.4 ± 56.3	<0.001^*^
The average intra-operative red blood cell transfusion (UI)	3.6 (3~5.5)	2.4 (2~3)	0.280
Blood type compatibility between donor and recipient [n (%)]	17 (94.44)	163 (95.32)	0.602
Surgical approach (piggyback liver transplantation) [n (%)]	16 (88.89)	160 (93.57)	0.357
GRWR (%)	2.7 ± 1.4	3.2 ± 1.1	0.076
Anhepatic stage (mins)	54.4 ± 12.2	49.4 ± 11.0	0.071
Tacrolimus steady-state concentration (ng/ml)	9.6 ± 2.3	9.2 ± 1.4	0.478
AKI stage 3 [n (%)]	15 (83.33)	19 (11.11)	<0.001^*^

*BA, Biliary atresia; GRWR, Graft to Recipient Weight Ratio; PELD, pediatric end-stage liver disease*.

* *P < 0.05, compared with the CRRT group*.

Comparisons of the postoperative complications are presented in Table 2. Compared with the non-CRRT group, the CRRT group had higher incidences of postoperative unscheduled surgery (8.19 vs. 44.44%, *P* < 0.001), postoperative primary nonfunction of the transplanted liver (0.00 vs. 5.56%, *P* = 0.002), secondary liver transplantation (0.00 vs. 16.67%, *P* < 0.001), hepatic artery occlusion (2.34 vs. 16.67%, *P* = 0.002), and intestinal fistula (1.17 vs. 11.11%, *P* = 0.005).

**Table 2:** Comparison of the postoperative complications in pediatric patients with acute kidney injury after liver transplantation.

**Items**	**CRRT group, *n* = 18**	**non-CRRT group, *n* = 171**	***P value***
Postoperative unscheduled surgery [n (%)]	8 (44.44)	14 (8.19)	<0.001^*^
Hepatic insufficiency [n (%)]			
Primary nonfunction [n (%)]	1 (5.56)	0 (0.00)	0.002^*^
Acute rejection [n (%)]	2 (11.11)	5 (2.92)	0.080
Drug-induced liver injury [n (%)]	1 (5.56)	4 (2.34)	0.419
Secondary liver transplantation [n (%)]	3 (16.67)	0 (0.00)	<0.001^*^
Infectious complications [n (%)]	6 (33.33)	34 (19.88)	0.184
Vascular complications [n (%)]			
Portal thrombosis [n (%)]	2 (11.11)	5 (2.92)	0.080
Hepatic artery occlusion [n (%)]	3 (16.67)	4 (2.34)	0.002^*^
Outflow tract obstruction [n (%)]	2 (11.11)	9 (5.26)	0.313
Biliary complications [n (%)]			
Biliary stenosis [n (%)]	0 (0.00)	7 (4.09)	0.382
Intestinal fistula [n (%)]	2 (11.11)	2 (1.17)	0.005^*^

* *P < 0.05, compared with the CRRT group*.

### Treatment of Continuous Renal Replacement Therapy in Children With Acute Kidney Injury

Of the 189 pediatric liver transplant recipients with AKI occurring between January 1, 2019, and June 1, 2021, 18 were treated with CRRT. These pediatric patients were all indwelled with a double-lumen catheter under ultrasound guidance. The main indwelling site was the left internal jugular vein (66.66%), followed by the right internal jugular vein (33.33%). Continuous renal replacement therapy was conducted a total of 59 times in the 18 patients. Among them, 25 (46.30%) involved continuous venovenous hemodiafiltration, 18 (33.33%) involved continuous venovenous hemodialysis, and 13 (24.07%) involved continuous venovenous hemofiltration. Among these 18 patients received CRRT, seven patients with AKI directly (0–7 days) after primary liver transplantation (38.89%), eight patients developed AKI later but <30 days after primary transplantation (44.44%), and three developed AKI later than 30 days (16.67%). The anticoagulation of the pipeline and filter was dominated by sodium citrate (59.26%), argatroban (25.93%), and sodium heparin (14.81%). The completion rate of CRRT was 96.61%, with only two treatments being terminated due to septic shock with circulatory instability. The median time to initiate CRRT was 10 days postoperatively. Pediatric patients who underwent unscheduled surgery started CRRT an average of 1.5 (0–15) days after their unscheduled surgery; the median number of CRRT treatments per patient was 2 (1–14), the median duration of each treatment was 10.1 (6–19.3) h, and the average rate of decrease in blood Cr per treatment was 25.6% (13.5%-45%). The therapeutic conditions in pediatric patients with AKI are illustrated in Table 3.

**Table 3:** Details of CRRT of each patient in the CRRT group.

**Groups**	**Case number**	**Gender**	**Age (Month)**	**CRRT date [post-operation (Day)]**	**Unscheduled surgery date [post-operation (Day)]**	**The name of unscheduled surgery**	**Times of CRRT**	**The average treatment duration per time (hours)/the total treatment time of CRRT (hours)**	**The average rate of decrease in blood creatinine per treatment (%)**	**Prognosis**
Group 1	3	Female	7	6	/	/	1	6/6	28	Improvement
Group 1	4	Male	6	4	/	/	2	6/12	19.5	Improvement
Group 1	6	Female	8	2	2	Secondary liver transplantation	4	6/24	15.4	Improvement
Group 1	8	Male	7	1	/	/	4	10/40	27.6	Improvement
Group 1	10	Male	4	3	/	/	2	10/20	16.7	Improvement
Group 1	13	Female	4	5	/	/	2	12/24	26.4	Improvement
Group 1	17	Female	5	6	1	Portal vein thrombectomy	10	16.5/165	28.6	Died of septic shock
Group 2	1	Female	8	10	/	/	2	8.5/17	23.2	Improvement
Group 2	2	Female	9	8	/	/	4	6/24	25.5	Improvement
Group 2	5	Female	5	15	14	Intestinal perforation repair	1	6/6	45	Improvement
Group 2	9	Female	8	10	9	Intestinal perforation repair	1	1/10	37	Improvement
Group 2	11	Female	5	10	8	Secondary liver transplantation	2	10/20	35.7	Improvement
Group 2	15	Male	12	14	10	Wound debridement	3	19.3/58	25.1	Died of sepsis, multiple organ failure
Group 2	16	Female	10	20	20	Exploratory laparotomy for hemostasis	1	10/10	13.5	Died of acute left heart failure
Group 2	18	Male	36	30	/	/	2	15/30	19.1	Died of massive gastrointestinal hemorrhage
Group 3	7	Female	8	60	/	/	2	10/20	33.1	Improvement
Group 3	12	Male	24	240	/	/	14	10.9/153	26.3	Improvement
Group 3	14	Male	12	60	45	Secondary liver transplantation	2	10/20	15.2	Died of antibody-mediated rejection

*Group 1: with patients developed AKI directly (0-7 days) after primary liver transplantation*.

*Group 2: with patients developed AKI later but <30 days after primary transplantation*.

*Group 3: with patients developed AKI later than 30 days after primary transplantation*.

### Results of Multivariate Logistic Regression Analysis of CRRT-Related Factors

Variables with significant differences in Tables 1, 2 were included in the multivariate logistic regression analysis. Finally, AKI stage 3 [OR=40.000, 95%CI (10.598, 150.969), *P* = 0.016], postoperative unscheduled surgery [OR=6.269, 95%CI (3.051, 26.379), *P* = 0.007], and hepatic artery occlusion [OR=17.682, 95%CI (1.707, 40.843), *P* = 0.001] were recognized as risk factors for postoperative AKI with CRRT therapy (Table 4).

**Table 4:** Results of multivariate logistic regression analysis of CRRT-related factors.

**Variable**	**OR**	**95% CI**	***P*** **Value**
The operation time of liver transplantation	4,165.502	3.552~418.537	0.962
The average intra-operative hemorrhage	3,286.377	2.659~282.642	0.706
AKI stage 3	40.000	10.598~150.969	0.016^*^
Postoperative unscheduled surgery	6.269	3.051~26.379	0.007^*^
Primary nonfunction	47,089.000	0.602~168.135	>0.999
Secondary liver transplantation	39,846.000	3.348~349.383	>0.999
Hepatic artery occlusion	17.682	1.707~40.843	0.001^*^
Intestinal fistula	9.416	1.393~80.098	0.054

*OR, Odds Ratio; 95%CI, 95% Confidence Interval; AKI, acute kidney injury*.

* *P < 0.05, with statically significant difference*.

### Survival Analysis of Infants and Children With Acute Kidney Injury After Liver Transplantation

**Figure 1:**
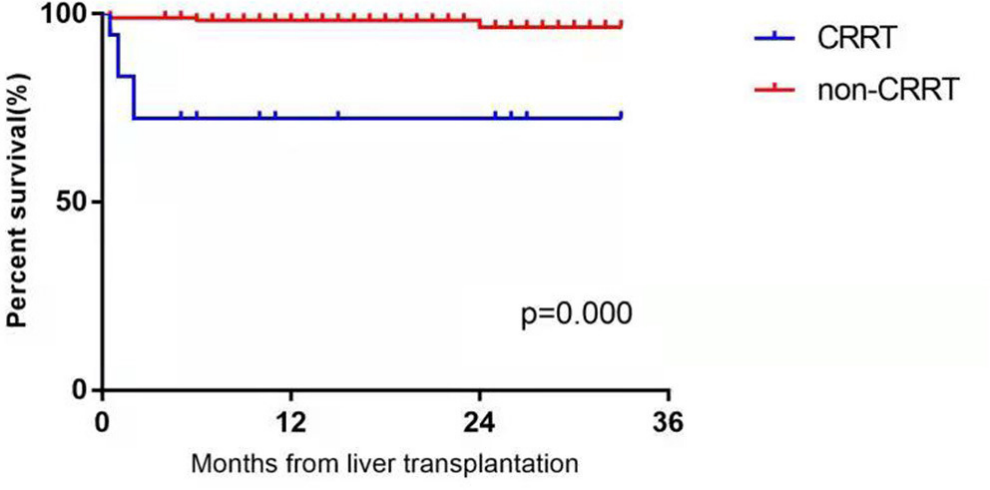
The postoperative survival curves in the CRRT group and non-CRRT group of infants and younger children with acute kidney injury after liver transplantation.

Until October 1, 2021, 189 pediatric patients with AKI were followed up for 4 to 33 months (average follow-up duration: 9.5 months). Thirteen of the children with AKI in the CRRT group improved, and five died. The causes of death were antibody-mediated rejection, sepsis, acute left heart failure, septic shock, and massive gastrointestinal hemorrhage. The time of death ranged from 13 to 58 days postoperatively. In the non-CRRT group, six pediatric patients with AKI died. The causes of death were abdominal infection 2 weeks after liver transplantation in two cases, septic shock 1 month after surgery in two cases, severe pneumonia 6 months after surgery in one case, and lymphoma 24 months after surgery in one case. The one- and two-year survival rates were 72.22% and 72.22% in the CRRT group, respectively, and 97.08% and 96.49% in the non-CRRT group, accordingly. There were statistically significant differences in the one- and two-year survival rates between the two groups (*P* < 0.001). The survival curves of the two groups are plotted in Figure 1.

## Discussion

Acute kidney injury is a common comorbidity in patients with chronic liver disease. Hypovolemia, infection, acute tubular necrosis, and hepatorenal syndrome are the leading causes of AKI in patients with chronic liver disease (3–6). Due to immature organ development in infants, their glomerular filtration function, renal tubular reabsorption, and concentration and dilution functions are lower than in adults, and they do not reach adult levels until after 2 years of age. Infants and young children have poor renal reserve functions and are more susceptible to AKI due to physical and chemical factors, including hypoperfusion, ischemia, hypoxia, and medications.

Acute kidney injury is also one of the most common complications after surgery. The kidney is an extremely sensitive organ to ischemia and hypoxia. When the body suffers massive blood loss and hypotension during surgery, the kidney is hypoperfused, urine volume decreases rapidly, and significant changes in renal function occur within a short period. The incidence of AKI is especially high in surgeries with large hemodynamic fluctuations and prolonged operating times, such as cardiac, aortic, and relatively larger abdominal surgeries.

Liver transplantation is the largest abdominal surgery, involving long operative times, massive intraoperative hemorrhage, and significant hemodynamic fluctuations. Acute intraoperative blood loss, hypotension, blockage/opening of the vena cava, the application of large amounts of intraoperative anesthetics and vasoactive drugs, and the release of intraoperative inflammatory factors may lead to the development of AKI (7). In a retrospective analysis of 198 pediatric patients with biliary atresia who underwent parental liver transplantation, Wu et al. (2) of the Department of Anesthesiology at Tianjin First Central Hospital found that the incidence of AKI after liver transplantation was 41.92%, low preoperative serum levels of Cr and high preoperative pediatric end-stage liver disease (PELD) scores were independent risk factors for the development of postoperative AKI, and the morbidity and mortality of pediatric patients in the AKI group (7.2%) were significantly higher than in the non-AKI group (0.8%). Dou et al. (8) conducted real-time intraoperative hemodynamic monitoring in 112 pediatric patients with parental liver transplantation and found that the incidence of AKI was 40.18%; furthermore, severe myocardial depression and significant decreases in the cardiac output and blood pressure in the early stages of reperfusion had an independent positive correlation with postoperative AKI. For pediatric liver transplant recipients, AKI is an independent risk factor for postoperative mortality (9–11).

Current studies on postsurgical AKI have focused on the influence of preoperative and intraoperative factors on the development of AKI, while less research has been conducted on the association of postoperative factors with the prognosis of AKI. Based on a previous study by the anesthesiologists at our hospital, a retrospective analysis of 512 infants and younger children with liver transplantation was conducted, focusing on the development of AKI after liver transplantation, the postoperative complications, and the prognosis of AKI in pediatric patients. The statistical results revealed that a total of 189 pediatric patients developed AKI within 1 year after surgery with an incidence of 36.91%, which was close to that reported in the literature. These patients were divided into CRRT and non-CRRT groups according to whether or not CRRT was conducted. There was no significant difference between the two groups in terms of preoperative PELD score, antimicrobial exposure, and the indwelling of a central venous catheter. In terms of intraoperative factors, the CRRT group had a longer operation time and a larger volume of intraoperative hemorrhage. In terms of postoperative factors, the incidence of postoperative unscheduled surgeries, primary nonfunctions of the transplanted liver, secondary liver transplantations, hepatic artery occlusions, and intestinal fistulas were higher in the CRRT group than the non-CRRT group (*P* < 0.05). Postoperative unscheduled surgery was caused mainly by serious complications, such as intestinal perforation, abdominal hemorrhage, portal thrombosis, nonfunction of the transplanted liver, etc. These resulted in abdominal infection, hypotension, anemia, acidosis, disturbance of the internal environment, sepsis, and even shock, which further aggravated AKI. It was noted that 8 of the 18 patients in the CRRT group had a second surgery after liver transplantation, and the median time to initiate CRRT for these patients was 1.5 (0 to 15) days after the second surgery. These results suggested that severe postoperative complications and the blow of a secondary operation would accelerate impaired renal function and drive CRRT.

Continuous renal replacement therapy is one of the most commonly used modes of blood purification. Yang Xue et al. (12) conducted a cross-sectional survey of CRRT in pediatric intensive care patients in 53 hospitals in 39 cities in China from 2012 to 2016. They found that CRRT was carried out in most regions and was the most frequently used blood purification technique, which was related to the broad indications for CRRT. Continuous renal replacement therapy can remove toxins and inflammatory small and medium molecular weight mediators, enabling its use in cases of simple renal failure, cardiac failure, volume overload, and the treatment of sepsis, MODS, ARDS, severe pancreatitis, and some immune diseases. Infection is often the first cause and the first-ranked postoperative complication in critically ill pediatric patients in medicine or surgery. Therefore, infection secondary to the impairment of organ function, i.e., sepsis, is also a common cause of CRRT. Despite relentless international and national battles against sepsis and the publication of numerous guidelines, the mortality from sepsis remains high (13). In the present study, among the patients who underwent CRRT due to AKI exacerbation, six cases were secondary to the ischemic–hypoxic shock of liver transplantation, three were secondary to systemic multi-organ functional impairment (including of the kidney due to nonfunction of the transplanted liver), and nine were secondary to sepsis due to severe primary infection resulting in renal involvement. Five of the 18 cases with CRRT died, and all were blown by the secondary operation. In exploring the causes of death (e.g., infection, rejection, left heart failure, or gastrointestinal hemorrhage), sepsis was the typical outcome.

Hemodialysis in infants and younger children is more challenging to implement and more difficult to maintain than in adults. First, the dwelling of deep vein catheters is more difficult in infants than in children or adults and usually requires an ultrasound-guided puncture. Second, circulation maintenance needs to be more refined. In general, the volume of extracorporeal circulation during blood purification should not exceed 10% of the total circulating volume; otherwise, drawing blood at the beginning of treatment may cause insufficient effective circulation and lead to the risk of shock (14, 15). Therefore, at the beginning of the treatment, normal saline containing albumin or 100 ml of plasma should be used to prefill the hemofilter and the vascular pipeline to ensure the return of the colloidal solution to the body when drawing blood. The parameters should be set according to the body mass of the pediatric patient during treatment, and their vital signs should be closely monitored to maintain circulatory stability. Third, in terms of the anticoagulation and maintenance of the filter, anticoagulants should be selected based on a comprehensive assessment of coagulation function, bleeding tendency, platelets, etc. In patients after liver transplantation, if the liver function recovers well, coagulation is in a recovery state, and there is no bleeding tendency, typically, sodium heparin or argatroban should be selected. If the patient has poor liver function, abnormal coagulation, or a significant bleeding tendency, sodium citrate should be a relatively safe choice (16–20). Fourth, the reserve capacity of the liver, kidney, and cardiopulmonary function in infants and young children is poor. Therefore, blood purification treatment should be timely, as treatment that is too late increases the difficulty of various procedures, including catheter indwelling and maintenance. Organ function might be irreversibly damaged, leading to circulatory failure and serious/persistent internal environmental disorders.

This study also has some limitations. Firstly, due to the limited cases, three patients who developed AKI and received CRRT later than 30 days after primary transplantation were included in this study, which may cause selective bias. In these cases, the main causes for pediatric patients to undergo CRRT may not be directly related to the primary transplantation which had been confirmed by the multivariate logistic regression analysis. So, it would be better to implement stricter inclusion criteria in the further study, only to include pediatric patients who developed AKI within 7 days and were treated with CRRT. Secondly, the number of patients in the non-CRRT group was nearly 10 times more than that in the CRRT group, which may decrease the test power. The best way was to include more pediatric patients who underwent CRRT with AKI after the liver transplantation. It would be better if the ratio of patient numbers between the non-CRRT group and the CRRT group was 5:1.

## Conclusion

The incidence of AKI after liver transplantation in pediatric patients was high. Patients with AKI stage 3, hepatic artery occlusion, and underwent unscheduled surgery postoperatively were with a high likelihood of receiving CRRT, which were related with a lower one- and two-year survival rates.

## Data Availability Statement

The original contributions presented in the study are included in the article/supplementary material, further inquiries can be directed to the corresponding author.

## Ethics Statement

The studies involving human participants were reviewed and approved by Tianjin First Central Hospital. The patients/participants provided their written informed consent to participate in this study.

## Author Contributions

Conception and design of the research and writing of the manuscript: YS and BW. Acquisition of data: SG, XW, LY, and MX. Analysis and interpretation of the data: SG and XW. Statistical analysis: MX and SG. Obtaining financing: BW and WG. Critical revision of the manuscript for intellectual content: LY, WG, and CS. All authors read and approved the final draft.

## Funding

This project were supported by Chunfeng Project of Tianjin First Central Hospital (2020CF05) and State Natural Science Fund Project (82170672).

## Conflict of Interest

The authors declare that the research was conducted in the absence of any commercial or financial relationships that could be construed as a potential conflict of interest.

## Publisher's Note

All claims expressed in this article are solely those of the authors and do not necessarily represent those of their affiliated organizations, or those of the publisher, the editors and the reviewers. Any product that may be evaluated in this article, or claim that may be made by its manufacturer, is not guaranteed or endorsed by the publisher.
